# To belong or not to belong: nursing students’ interactions with clinical learning environments – an observational study

**DOI:** 10.1186/s12909-016-0721-2

**Published:** 2016-08-05

**Authors:** Matilda Liljedahl, Erik Björck, Susanne Kalén, Sari Ponzer, Klara Bolander Laksov

**Affiliations:** 1 Department of Learning, Informatics, Management and Ethics, Karolinska Institutet, SE-171 77 Stockholm, Sweden; 2 Clinical Genetics, Karolinska University Hospital, SE-171 76 Stockholm, Sweden; 3 Department of Molecular Medicine and Surgery, Karolinska Institutet, Karolinska University Hospital, SE-171 76 Stockholm, Sweden; 4 Department of Clinical Science and Education, Södersjukhuset, Karolinska Institutet, SE-118 83 Stockholm, Sweden; 5 Department of Education, Stockholm University, SE-106 91 Stockholm, Sweden

**Keywords:** Belongingness, Clinical learning environments, Clinical nursing education, Observational study, Workplace learning, Workplace participatory practices

## Abstract

**Background:**

Belongingness has been argued to be a prerequisite for students’ learning in the clinical setting but making students feel like they belong to the workplace is a challenge. From a sociocultural perspective, workplace participatory practices is a framework that views clinical learning environments to be created in interaction between students and the workplace and hence, are dependent on them both. The aim of this study was to explore the interdependence between affordances and engagement in clinical learning environments. The research question was: How are nursing students influenced in their interactions with clinical learning environments?

**Methods:**

An observational study with field observations and follow-up interviews was performed. The study setting comprised three academic teaching hospitals. Field observations included shadowing undergraduate nursing students during entire shifts. Fifty-five hours of field observations and ten follow-up interviews with students, supervisors and clinical managers formed the study data. A thematic approach to the analysis was taken and performed iteratively with the data collection.

**Results:**

The results revealed that students strived to fill out the role they were offered in an aspirational way but that they became overwhelmed when given the responsibility of care. When students’ basic values did not align with those enacted by the workplace, they were not willing to compromise their own values. Workplaces succeeded in inviting students into the community of nurses and the practice of care. Students demonstrated hesitance regarding their desire to belong to the workplace community.

**Conclusion:**

The results imply that the challenge for clinical education is not to increase the experience of belongingness but to maintain students’ critical and reflective approach to health care practice. Additionally, results suggest students to be included as an important stakeholder in creating clinical learning environments rather than being viewed as consumer of clinical education.

**Electronic supplementary material:**

The online version of this article (doi:10.1186/s12909-016-0721-2) contains supplementary material, which is available to authorized users.

## Background

The clinical nursing education literature emphasises students’ experience of belongingness to the workplace as a prerequisite for learning [1]. It has thus been suggested that becoming a nurse includes membership and participation in a community of practice of nursing [2, 3]. Being part of an authentic setting makes learning real as students are able to interact with patients rather independently from their supervisors [4]. However, making students feel that they belong to a workplace is easier said than done. Conversely, the healthcare environment has been questioned as being favourable learning environments due to, e.g. increasing work load and the challenge of organising supervision [5, 6]. Further, clinical placements have been characterised as a source of stress for students, especially when facing moral dilemmas [7]. Regarding nursing students’ experiences of clinical learning environments, the supervisor relationship seems to be as crucial as it is challenging [3, 5, 6, 8]. Clinical placements are however widely recognised as essential for nursing students’ development into professional nurses. In order to utilise learning opportunities in the clinical setting, the complexity of clinical learning environments needs to be better understood. There is a vast body of literature in health sciences education investigating students’ perceptions about learning environments with instruments such as the Clinical Learning Environment Inventory and Clinical Learning Environment and Supervision [9, 10]. In this discourse, clinical learning environments are addressed as something students can perceive and are therefore understood as measurable [11]. However, from a sociocultural perspective on learning, clinical learning environments can be understood as being constructed in interactions between students and environments and, therefore, a more situational and dynamic feature than in the former discourse.

Billett’s [12] concept of ‘workplace participatory practices’ offers a framework for understanding learning in the workplace as an interdependent process between workplace affordances and individual engagement. In this framework, the workplace offers certain affordances for learning, that is, the available activities and how the workplace invites learners to participate in these activities. Access to participation is guided by workplace values and norms that build on workplace history and negotiated in interactions between established members of the workplace. Individual engagement refers to how individuals elect to engage in afforded activities and is arguably guided by personal values and history as well as individuals’ agency to engage in the workplace. Agency here relates to how individuals find meaning in participating in the available activities. For example, an experienced lack of relevance might decrease the likelihood of students’ engagement in a workplace. The potential educational value embedded in available activities might therefore not be fully utilised, if learners neglect to acknowledge them as pedagogical rich [13].

Clinical learning environments are arguably created in interactions between the workplace and individuals as the workplace affords learning opportunities in which individuals can elect to engage [14]. The concept of workplace participatory practices thus adopts a bi-directional approach to workplace learning whereby both the workplace and the students are viewed as agents and stakeholders [15]. Further, workplace participatory practices acknowledge the relationship between the workplace and students to be highly relational and interdependent [16]. Learning in the workplace can from this perspective be viewed as a relational and social act, dependent on both workplaces and students and therefore, complex by nature [14].

Applying the framework of workplace participatory practices to nursing students’ clinical education can offer insights into how they become participating members of a clinical workplace. Importantly, acknowledging both the workplace’s ability to invite students and their own agency to engage in the workplace can help us understand how the process of becoming a participant occurs in practice. However, as clinical environments have a major impact on students [3, 17, 18], one can reasonably assume that the workplace exercises greater levels of influence on students than vice versa. The students are thus at the centre of attention in this study.

The aim of this study was to explore the interdependence between affordances and engagement in clinical learning environments of nursing students. We posed the following research question: How are nursing students influenced in their interactions with clinical learning environments?

## Methods

### Context

The study setting comprised three publicly funded academic hospitals in Stockholm county council, Sweden, where nursing students from different universities had their clinical placements. The three-year undergraduate nursing programme in Sweden, which leads to a bachelor’s degree in nursing and registration as a nurse, combines theoretical courses with clinical placements. Students attend up to six placements during the programme and placements last approximately four-six weeks. Usually students have two or more supervisors during the placement period. Many supervisors are given the opportunity to take educational courses to develop as supervisors and clinical education managers. Educational and pedagogical issues at the workplaces have usually been extensively discussed, with several supervising models being sampled before agreeing on what was perceived as the most suitable one. As each clinical workplace decided on the supervisory model, the enacted one varied in-between clinical departments. In addition to the supervisors, a clinical education manager is responsible for overall administration and communication with teachers. In the mid- and end points of each placement, a university teacher and the supervisor assess students as per the intended learning outcomes of the placement. In the Swedish context, students actively participate in clinical practice and are sometimes given one or a few patients to care for under supervision.

### Design and theoretical framework

We designed an ethnography-inspired qualitative study [19], with data collected through field observations and follow-up interviews. As we drew upon a constructivist interpretative tradition, knowledge was viewed as relative and socially constructed [20]. Furthermore, we adopted a socio-cultural perspective on learning, meaning that learning was viewed as situated in a social world, dependent on individuals and the larger community [21].

### Procedure

In line with our philosophical orientation, data collection and analysis was an iterative process meaning that initial analysis of data guided further data collection. Further, data collection was guided by case study methodology which is understood to be especially beneficial when the boundary between the phenomenon and the context is unclear [22]. Accordingly, we strived for various settings in terms of medical specialty and hospitals. Also, we actively deselected wards designed exclusively for students as we suspected their interdependence between workplace and students to be of another nature then in the regular hospital setting.

**Fig. 1:**
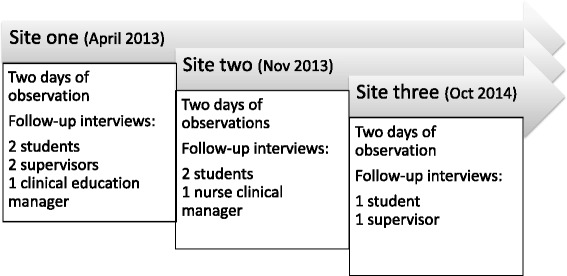
Overview of data collection. Data was collected at three sites where field observations were followed by follow-up interviews with some of the participants. As data analysis was performed iteratively with data collection, there is some time in-between the collection of data at each site

Data was collected from three sites during 2013 and 2014, one site at each hospital (Fig. 1). The sites were purposefully recruited through negotiations between the research team and gatekeepers [23], and approval to undertake ward observations was obtained from the head of each clinical department. The sites were diverse in terms of medical specialty but all held great educational responsibilities both on the undergraduate and postgraduate level. At each site, students with placements at the time of the planned data collection were asked by their supervisor or clinical education manager if they were interested in participating in the study. In the researchers’ interactions with both gatekeepers and potential participants, it was clarified that the intention of the study was not to assess clinical education but to explore the clinical environment with all its opportunities and challenges. In this way, the research team was able to build trust with the sites [24] as the observations were hopefully not perceived as evaluative. In addition, this approach might have reduced the observers’ effects on participants as they felt comfortable with not being assessed during observations [19]. All students who were asked agreed to participate in the study.

The first author (ML) performed non-participant observations [25], shadowing six students during an entire shift (approximately 9 h) and observing students’ activities and interactions. Even though only six students were shadowed, the observer came across several more during observations. Extensive observational and reflective field notes were taken [26]. During observations, ML wore a clinical uniform and introduced herself as ‘a medical student and researcher’ but did not interact with participants or others if they did not explicitly address her. Follow-up interviews with students and supervisors were performed preferably the same day as observations. In a few cases this was not possible due to practical reasons, and the follow-up interview was instead held a few days post observation. Follow-up interviews adhered to an interview guide based on the literature (covering aspects such as relationship to supervisor/student, experienced stress, and workplace culture), the research question and the present observation (Additional file 1). Follow-up interviews were audio recorded and lasted in average 20 min. During the observations, ML also approached other members of the workplace, holding informal interviews with them. Informal interviews were not recorded. ML instead wrote down the main messages from these interviews.

Altogether, the study data consisted of six days of observation (55 h) and ten interviews. All observations and interviews were transcribed verbatim, resulting in approximately 60,000 words of data. The analysis took an inductive thematic approach [27, 28] and was performed in the following steps:

1. An initial analysis was performed after collecting data at the first two sites. Influenced by critical incident analysis [29], ‘significant events’ were used as a tool for the initial analysis [30]. The first author identified seven significant events from field notes on a specific incident and excerpts from interviews detailing the incident. A significant event was understood as a situation involving, e.g. something emotional for the student, a conflict of any kind or in interviews with students or supervisors identified by them as a valuable learning situation.
2. Based on the analysis of the significant events, preliminary themes were identified.
3. All the data was arranged into a framework of intended (formally structured), incidental (unplanned and unscripted) and cultural (influences of an invisible character) learning activities adopted from the work of Hafler [31].
4. The arranged data was subsequently organised in categories within each type of learning activity (intended, incidental and cultural).
5. The previously identified themes were adjusted according to the categories and were based on the analysis of the entire dataset.
6. After the data collection at the third site, the analysis was finalised through discussions in the research team where themes were critically discussed, adjusted and analysed in relation to the literature.

This study is part of a PhD project and the interdisciplinary research team consisted of a diversity of perspectives, including nursing, medicine and education. The entire research process was characterised by continuous and critical discussion on design and analysis, both within the research team and in an extended network of researchers with backgrounds including medicine, nursing, higher education and social science. This was helpful throughout the research process and guided decisions on data collection, analysis and interpretation.

## Results

Three themes relating to how the interdependence between affordances and engagement influenced nursing students were identified (Table 1). The themes concerned different dimensions of clinical learning environments, the clinical learning community, the clinical learning design and clinical learning context. The dimension of each theme will assist in outlining what was apprehended about the theme. We shall now present the three themes with illustrations from field note excerpts and interview quotes.

**Table 1:** Overview of the results

Themes on how nursing students are influenced by clinical learning environments	Aspect of		Dimension of clinical learning environments
	Workplace affordance	Individual engagement	
Being aspirational in taking up the offered role	Offered conditional membership	Striving to fill out the role	Community of clinical learning
Being overwhelmed by the responsibility of care	Entrusted to provide care	Trying to handle the responsibility	Design of clinical learning
Being hesitant to negotiate own values with reality	Exposure to pragmatic reality	Challenging basic values	Context of clinical learning

Overview of results according to the three themes; aspects of workplace affordance and individual engagement; the dimensions of clinical learning environments in relation to the themes

### Theme one: Being aspirational in taking up the offered role

Supervisors provided space for students in the workplace, making them a natural and desirable part of the health care team. This meant that being new or being a beginner was accepted even though activities might take longer than with professional nurses. The staff addressed students who seemed unsure and would discretely guide them in terms of how to behave or act in different situations. Students were actively included in ward traditions and introduced to routines; for example, taking control of patients was supposed to be performed at specific intervals.

Two students are planning a shift together with their supervisors.

Supervisor one:

*The first 24 h, you’re supposed to take vital parameters every 8 h, but perhaps you do not need it in the ‘to do’ list?*

Supervisor two:

*Actually, I usually do that since the ward’s statistics are poor.* (Field note, 2^nd^ Year students, site one)

In this way, the workplace shared its norms and values with the students, for instance, that the ward’s goals for alignment with vital parameter routines were prioritised by the nurses. Students were invited into the professional community; however they were expected to align with the norms. In that sense, the offer of membership seemed conditional.

For students, it was important to perform tasks in the right way according to ward routines. For example, a student could stay awake at night wondering whether she had performed her tasks properly.

In the morning, a student poses questions arising from the previous night. Supervisors and students laugh together with the student at the fact that she could not sleep due to her clinical placement.

Student:

*What was it… the bag to the urine catheter, I drained it yesterday. Am I supposed to tell anyone about that?*

Supervisor:

*No, you do not have to tell anyone, but you can always do that twice during your shift. As a habit, you can do it during the nursing round.*

Student (laughing):

*OK, how lucky; I thought about calling the ward to tell them…* (Field note, 2^nd^ Year students, site one)

This field note highlights how the student, besides worrying about the patient, also seemed anxious about whether she had done everything right. Likewise, students were eager to be able to answer patients’ questions, perform procedures smoothly and remember all the facts about the patient. Students thus strived to fill out expected roles as they entered the workplace community.

*I sometimes wonder, perhaps obsessively, whether I’ve done everything right. Have I removed the IV-fluid on this patient; did I…? Sort of like, did I blow out the candles… and the worst thing I can keep on droning on is whether I’ve forgotten to say something.* (Interview, 2^nd^ Year student, site one)

In this theme, students thus demonstrated an ambition to manage the role of a professional nurse, taking it up in an aspirational way.

Student:

*I don’t like the feeling of insecurity. I’m the kind of person who prefers to know everything precisely before I do something myself.* (Field note, 2^nd^ Year student, site two)

### Theme two: Being overwhelmed by the responsibility of care

The workplace offered a clear structure of clinical education as students’ learning was taken into consideration in conjunction with ward routines. Students’ role, responsibility and progression throughout the placement were thought through by the workplace, and this usually meant that students were given responsibility and a mandate to care for patients. By giving students responsibility, the workplace endeavoured to create an authentic environment for them to practice away from the supervisor. Students were entrusted to receive notifications, and supervisors ensured that students had sufficient time to, e.g. come up with an action plan.

*It puts more responsibility on the students since they need to take initiative and do things… and experience responsibility themselves. They do not only follow and imitate us; they also reflect for themselves.* (Interview, supervisor, site one)

As such, the workplace entrusted students to provide care for patients. Students grasped the responsibility they were afforded and appreciated being entrusted even if they identified themselves as novices and unknowledgeable:

*There’s a lot that I still cannot manage. Practically, I don’t know anything at all (laughs). But still, I am given a lot of responsibility here. (…) And I am entrusted despite only being in my second year.* (Interview, 2^nd^ Year student, site two)

Even if students were allowed to interact independently with patients, supervisors clearly demonstrated full responsibility for patients by being available for students at all times and trying to make them feel safe. In the following situation, a student is about to perform a procedure on a patient:

The student explains to the patient what is about to happen. She is wearing gloves and an apron, as is appropriate for bedside working. The supervisor is present, standing next to the wall, but without gloves or apron. She looks relaxed, does not oversee the student and does not appear perturbed. The student starts but hesitates as the patient experiences pain. She looks to her supervisor for help, who immediately fetches gloves and approaches the bed to help out. (Field note, 2^nd^ Year student, site two)

For students, responsibility for patients seemed to be an energy-intensive assignment. It implied dealing with many issues simultaneously, which sometimes resulted in frustration from perceiving themselves as being slow. That they would eventually have to manage several patients concurrently was unimaginable to them.

*I feel like I’m perfectly occupied with two patients. That’s good enough. I wouldn’t want more.* (Interview, 2nd Year student, site two)

Students engaged in their patient’s well-being and appreciated how the relationships they were able to build with them could motivate their learning. They took responsibility for patients as they were eager to develop the capability to deliver care in a patient-safe manner. However, during independent activities with patients, students seemed to be fearful of failure, assessments and not knowing things they would have liked to know:

Two students are having a brief discussion during the intense morning shift:

The first student: *‘How many patients do you have?’*

The second student: *‘I only have one’.*

The first student: *‘I have two, and I get totally confused. Did I serve breakfast to the right person?’* (Field note, 2^nd^ Year students, site one)

As in this situation, students could at times easily forget and become confused over seemingly easy tasks. Even with few tasks and arguably enough time to solve them, students gave the impression of being stressed. Patient care was thus an exhausting experience, and students acted overwhelmed by the responsibility of care.

### Theme three: Being hesitant to negotiate own values with reality

The workplace made it possible for students to experience a ‘real’ clinical setting. In the clinical reality, students participated in ward activities and experienced the every-day complexity of the clinical ward. This could entail finding themselves stuck between the patient and the doctor as patients could have one view of their disease and the doctor an antithetical one. For example, some hospitalisations might seem unnecessary from the nursing perspective meaning that doctor’s decision could be understood as incomprehensive. From various members of the staff, patients were occasionally met with scepticism as they presented with conditions that the health care team found difficult to address:

A nurse from the night shift is handing over to the day team, which includes two nurses and a student.

One nurse: *‘But what caused the dizziness?’*

The other nurse: *‘Well, the patient thought it was because he was so angry with the home care service’.*

The nurses laugh. One nurse puts her hands to her head and pretends she is both angry and dizzy. The student notices her charade but fails to smile. (Field note, 2^nd^ Year student, site two)

In this field note, the nurses are making fun of the patient’s explanation of his symptom. Here, the workplace provided an example for students of how nursing could be put into practice, in a pragmatic and viable way, building on the ward’s extensive patient management experience.

Students approached their future professional role meaningfully and demonstrated high ambitions and values regarding patient treatment. Whereas supervisors responded to patients’ calls as time permitted, students immediately sought to help their patients.

A student helps a patient to the toilet, leaves the patient there and returns to make the patient’s bed. Upon finishing, he goes out into the corridor. The alarm system goes off, and the student realises that it is his patient; he immediately freezes in the corridor, turns around and promptly goes towards the toilet to help his patient. (Field note, 2^nd^ Year student, site one)

Students highly prioritized their patients in a way that was possible due to their workload but also in a way they viewed as necessary for high quality patient care. Accordingly, students sometimes reacted with deep concerns over actual patient treatment, at times strongly disagreeing with supervisors or other health care staff members’ assumptions about patients. Notwithstanding, they seldom spoke up, opting instead to express their disagreement to fellow students.

A student tells her peer students about a patient encounter when another member of the staff had expressed his mistrust in the patient’s symptoms:

*The patient said: ‘Not being able to find your own feet is really hard’. But it is tough as this is the first time I am meeting her, and I have my view of her, and then he [a staff member] comes and expresses his view. But I still have my view, you know…* (Field note, 2^nd^ Year students, site one)

In this field note, the student reflects upon a patient encounter where the student disagreed with a member of the staff. This time the student did not speak up. The following discussion in-between the peers went as follows:

A student: Perhaps we should ask them [why they did like that]?

Second student: Sure, but then you’re a student and do not dare.

Third student: Next week perhaps… We might dare then. (Field note, 2^nd^ Year students, site one)

In this way, the students confronted their own basic values regarding patient care with the reality of the workplace and, even though not openly communicated, they demonstrated hesitance regarding their willingness to adjust to the workplace culture. One student puts it like this:

*During reporting, they make comments like: ‘This patient has already returned! She was here last week!’ Everyone sighs. And I’m thinking ‘is this really necessary?’ […] These preconceptions that one can have about patients… I never want to be like that.* (Interview, 2^nd^ Year student, site one)

## Discussion

This study explored clinical learning environments of nursing students from a workplace participatory practices perspective. We identified aspects of workplace affordances and individual engagement and analysed interdependent interactions between students and the workplace. This resulted in three themes on how nursing students are influenced in their interactions with clinical learning environments.

The first theme implied that students were aspirational in their efforts to take up the roles the workplace afforded them. Our results suggest that the membership offered to students at the workplace was conditional. While students felt welcomed and included, the workplace implicitly expected them to align to ward routines and traditions. Importantly, neither supervisors nor managers advocated for conditional membership and worked arduously to include and invite student, making them feel at home and safe on the ward. The importance of an inclusive approach to students during clinical placement has been variously highlighted [3, 32]. However, joining a community of practice involves negotiating with existing members even if newcomers tend to initially align to workplace norms to gain acceptance in the community [33, 34]. Thus, belonging to the group can be of greater importance than following one’s own values. In this study, students endeavoured to fill out the roles offered in an aspirational way. Noteworthy, in this theme, students sought to fulfil their roles not only in relation to patients but also in relation to the community of nurses. Consequently, students were eager to perform tasks in the right way, which meant that they were exposed to the boundaries of the local practice. It was as if the workplace was saying: *if you do not practice the way we do here, you cannot belong to our community*. Students thus sought to align to the expectations of the workplace. One can imagine that the fear of failing to fill out a role can hinder learning, the precise finding in a study by Levett-Jones et al. [3], is that students who experience support from their supervisors could focus on learning instead of being preoccupied with staff relationships. Further, Manninen et al. [4] advocate that when students’ experience belongingness to a team, they are able to focus on learning and understanding nursing. Moreover, from a sociocultural perspective, insufficient experience of belongingness to the community will likely deter learning [33, 35]. Indeed, including students in workplace communities continues to be crucial for student learning [36] as they, to some extent, will need to align to the workplace culture. Nevertheless, it is important that they maintain their critical and reflecting approach in order to develop their own professional identity and not simply adjust to existing ones [37].

The second theme entailed students becoming overwhelmed by the responsibility of care. The results suggest that students were afforded responsibility in line with the educational framework of clinical education and sought to handle the responsibility for patients. Participating in practice is in line with contemporary educational theories emphasising opportunities for learners to be active as a central feature of learning, e.g. learning-as-participation [38], transformative learning [39] and the theoretical framework of this study, workplace participatory practices [12, 14]. Here, the workplace succeeded in involving students, making them active in both practice and their own learning, which students previously have highlighted as fundamental to learning environments [17]. Independently caring for patients was, however, seemed to be an exhausting and challenging experience for nursing students, as previously described in the study by Manninen et al. [4]. In the current study, students were occasionally so overwhelmed by the responsibility of care that they for example could forget which patients had been served breakfast. Their capability to deal with extensive workloads can, in this phase of their training, be regarded as limited as students, in this study, could experience stress even during calmer shifts. Given that students are expected to develop from novices into professional nurses within three years, being overwhelmed is perhaps unsurprising. It might therefore be of importance for clinical education to facilitate students’ development of the capability of handling responsibility. McNamara reported on a highly appreciated programme aiming to prepare students for clinical placements including role-plays to simulate clinical processes from admission to discharge [40] which might be one way of exposing students for responsibility early. Interestingly, Odland et al. showed that newly qualified nurses were overwhelmed by the responsibility as they had not been experiencing that as nursing students [41]. Clinical education studied here seems in that sense to be successful in preparing students for transition into the professional role as they already as undergraduates experienced responsibility. It is interesting to notice here, that students did not show any indications of being abandoned. On the contrary, supervisors were present and accessible for questions and support. We believe that this availability was significant for students’ opportunities to practice responsibility in a safe environment.

The third theme indicated that students became hesitant regarding their willingness to negotiate their own values with the experienced reality. In a meta-study of nursing socialisation, Price [18] found ‘the paradox of caring’ as a salient theme connoting students’ struggle with conflicting notions of nursing. Poor role models who do not reflect students’ ideals are known to be a common feature of clinical education [42], however, role models are also known to be of strong influence for nursing students [3, 18]. Manninen et al. [43] found that nursing students become increasingly self-centred towards the end of their training, arguing that previous experiences of poor role models might explain this tendency. Conversely, the students in this study did not demonstrate complete alignment to workplace practices but, instead, resistance to what they saw as poor patient care. Coherent and strong ideals were found among nurses on qualification in a previous study, which support our results [44]. In fact, students’ hesitance to adapt to the existing reality might be an indication of their critical and reflective approach. However in the aforementioned study, the majority of participants could only two years after graduation be described as “compromised” or “crushed” idealists [44]. This could of course be a reflection of today’s health care; withstanding your ideals is impossible with the workload and reality of nursing. However, as mentioned before, making students and newly graduates maintain their reflective approach is central for them not to abandon their ideals.

In terms of belongingness, research shows that interactions within learning environments heavily determine the extent to which student feel included and accepted [1, 3, 36]. Positive student-staff relationships are understood to improve student perceptions of their clinical learning environments [3]. However, the present results suggest that students might not have a desire to belong as this would require them to compromise their own values regarding patient care. Thus, belongingness to the workplace and membership in the community were not dependent only on the workplace’s ability to include students but also on students’ willingness to engage in the workplace. From a workplace participatory practices perspective, workplace learning is understood as dependent on both the affordances of the workplace and the engagement of individuals, in this case, students [12, 15]. It might be the case that the impact of students’ individual engagement when considering learning environments has been underestimated. As attention has been on workplace functions and supervisors’ actions, the most important stakeholders in clinical education, namely, students have been omitted.

In relation to practice, the present results further establish the notion that nursing students are assisted in their learning by being invited into the community and given responsibility for patient care. The combination of these factors provides students with favourable opportunities for learning. In addition, exposure to the reality of health care enables authentic learning and knowledge transferability to the context in which students will eventually practice [10]. This study highlights the need to acknowledge students as an important stakeholder in creating clinical learning environments rather than viewing them as consumers of the environments. We therefore argue that the concept of workplace participatory practices, as outlined by Billett [12, 15], can be suitable in further exploring clinical education as it recognises both workplace affordances and individual engagement.

We argue that the three themes concern different dimensions of clinical learning environments; the learning community, the learning design and the learning context. The *learning community* includes the role students are given and concerns how newcomers are made members of the workplace community. The *learning design* entails how activities are structured, planned and introduced to facilitate learning and relates to the educational framework of the workplace. The *learning context* involves the setting in which learning takes place and the contextual factors that impact and influence learning. Due to its established significance, clinical learning environments have been extensively researched, but theoretical perspectives on learning environments remain scarce [45]. The educational environment is arguably a multi-layered, multi-dimensional phenomenon that is not easily grasped or measured [11]. Our results suggest that clinical learning environments contain at least three dimensions: community, design and context. Further empirical work is needed to investigate the stability of these dimensions, extending the list if necessary.

### Methodological limitations

As with all scholarly work, this study has some limitations. ML was a novice observer, and despite the methodological support she received from the research team, it potentially influenced the quality of the data collected. Our decision to collect data at three different sites limited the possibility to engage deeply in a single context. At the same time, dependability was enhanced as themes displayed consistency over the three sites. Moreover, in terms of credibility, the three sites also provided us with various perspectives on the phenomenon. There might be a risk that field observations influence behaviours of participants, referred to as the observer effect [46]. In our case, the study objectives were made clear for participants, limiting potential experience of being assessed. Additionally, as the observer was a student herself, she might have been perceived as less threatening. However, as students were asked to participate by their supervisor, they might have felt obligated to take part in the study.

While field observations and interviews were conducted by a single investigator, the analysis was collaboratively performed. As the research team comprised a variety of perspectives, including nursing, medicine, education and sociology, the research process was characterised by an open-minded and critical approach to the results and its relation to previous research and theory.

Although this study drew inspiration from the ethnographic tradition, we do not consider it to be an ethnographic study, which would have required more extensive field observations [19]. Field observations might be criticised as subjective and lacking continuity; however, it can be argued that when performed properly, they can contribute to understanding social phenomena [47]. Although field observations are often performed at a limited number of sites, researchers often find that sites share more commonalities than differences, implying that the results are likely transferable to similar contexts [48], which we believe was the case also in this study. The provided details on context and procedures, as done in this study, might also increase readers’ opportunities to transfer the results to their own settings.

## Conclusion

This study explored clinical learning environments of nursing students from a workplace participatory practices perspective. Workplaces succeeded in inviting students into both the community of nurses and the practice of patient care. However, the main message from the results was that nursing students negotiated their belongingness with the community because of strong inherent norms and values in the profession. In this negotiation, students showed hesitance regarding compromising their own basic values with the experienced reality of health care. Consequently, the challenge for clinical nursing education is perhaps not to increase students’ experience of belongingness to the workplace but to maintain their critical and reflective approach to health care practice. Centring on the initial question ‘to belong or not to belong’, Hamlet himself concludes: *‘Thus conscience does make cowards of us all, and thus the native hue of resolution, is sicklied o’er with the pale cast of thought’.* Upon closer examination, it appears necessary to upgrade the student as a stakeholder in creating, rather than being a consumer of, clinical learning environments. If students do not have the desire to belong, no inclusion whatsoever will incorporate them as members of the workplace. This study highlights the significance of students’ individual engagement in their interactions with clinical learning environments.

## Additional file

Additional file 1: Interview guide. Interviews adhered to the following interview guide. (DOCX 22 kb)
