# Supplementary material for: To belong or not to belong: nursing students’ interactions with clinical learning environments – an observational study
Source: BMC Med Educ. 2016 Aug 5;16:197. doi: 10.1186/s12909-016-0721-2 (PMC4974733; doi:10.1186/s12909-016-0721-2)
Supplement: Additional file 1: — Interview guide. Interviews adhered to the following interview guide. (DOCX 22 kb) [file 12909_2016_721_MOESM1_ESM.docx]

**Interview guide**

Presentation of the interviewer and the research project. Information regarding informed consent. Contact details to principal investigator.

Starting questions

- I have been shadowing you for a couple of days. Is there anything in particular that has come to your mind during this time?
- How have you experiences this placement?

Goal/Motivation

- Why do you participate in clinical placement? What is the benefit for you, personally?
- What expectations did you have before starting this placement? Have they been met?
- What goals did you have for yourself, starting this placement?
- What keeps you continuing this rotation?
- What have you appreciated the most with this placement?

Organisation

- How well does this clinical department seem to work?
- How were you receives as students?
- What kind of attitude do supervisors have towards students? How is it noticed?
- Who seems to be in charge here?
- If you compare with your previous placements, what similarities and differences have you been noticing?

Learning

- What are you supposed to learn here?
- What strategy for learning do you have?
- Have you learnt what you wanted to learn?
- What has been the most difficult here?

Observations: Come back to some situations noticed during observations

(Write down a few to take up in the interview)

- Can you describe this situation? What was happening there?
- What in this situation made you learn something?
- What prerequsites were in place?

Workplace culture

- How would you describe the atmosphere among staff here?
- How is the atmosphere towards you as students?
- Do you feel like you are making a difference for patients?

Conclusion

- What are you main take home messages from this placement
- Did you experience anything you would consider as dysfunctional? What could be made about that?

Thank you for participating in this study
